# 血清miR-19a-3p在多发性骨髓瘤诊断及预后评估中的作用研究

**DOI:** 10.3760/cma.j.issn.0253-2727.2022.07.012

**Published:** 2022-07

**Authors:** 晓晶 魏, 兰婷 刘, 浩 孙, 录贵 邱, 牧 郝

**Affiliations:** 中国医学科学院血液病医院（中国医学科学院血液学研究所），实验血液学国家重点实验室，国家血液系统疾病临床医学研究中心，细胞生态海河实验室，天津 300020 State Key Laboratory of Experimental Hematology, National Clinical Research Center for Blood Diseases, Haihe Laboratory of Cell Ecosystem, Institute of Hematology & Blood Diseases Hospital, Chinese Academy of Medical Sciences & Peking Union Medical College, Tianjin 300020, China

多发性骨髓瘤（MM）是一种以浆细胞克隆性增殖为特征的血液系统肿瘤，其典型的临床症状为高钙血症、贫血、肾功能损伤以及骨髓瘤骨病[1]。MM肿瘤细胞具有多灶性分布、肿瘤细胞异质性强的特点。因此在临床检测中基于单一位点的骨髓穿刺活检并不能全面评估肿瘤的细胞生物学特征。

miRNAs分子是一类小分子RNA，通过转录后水平调控基因表达。研究发现，肿瘤细胞具有特异的miRNAs表达谱，参与肿瘤的发生发展[2]–[4]，且是抗肿瘤治疗的潜在靶点[5]。近年来研究显示，miRNAs可稳定存在于血液、尿液、脑脊液等多种体液中，是一种全身性生物标志物，可用于疾病诊断、疗效评估[6]–[8]。本研究组前期研究报道，血清miRNAs在MM患者中表达失调，可用于MM的辅助诊断和患者的预后评估[9]–[10]。随着MM新药及新一代蛋白酶体抑制剂、免疫调节剂的应用，MM患者的生存时间明显延长[11]。本研究探讨了在新药时代，血清miR-19a-3p水平是否仍可作为MM患者诊断及预后评估的生物标志物。

## 病例与方法

1. 病例资料：本研究纳入2014年1月至2017年4月中国医学科学院血液病医院淋巴瘤中心收治的201例初诊MM（NDMM）患者，其中男119例（59.2％），女82例（40.8％），中位年龄59（34～84）岁，ISS分期Ⅰ期40例（19.9％），Ⅱ期69例（34.3％），Ⅲ期92例（45.8％）。R-ISS分期Ⅰ、Ⅱ、Ⅲ期患者分别为24例（11.9％）、123例（61.2％）和54例（26.9％）。细胞遗传学异常中del（13q）94例（46.8％），del（17p）21例（10.4％），t（4;14）31例（15.4％），1q21+ 98例（48.8％）。133例（66.2％）患者接受基于蛋白酶体抑制剂的一线治疗，50例（24.9％）患者接受基于免疫调节剂的一线治疗，28例（13.9％）患者接受了自体造血干细胞移植（auto-HSCT）（年龄≤65岁），仅4例（2％）患者未接受任何药物治疗。收集60名健康供者（HD）的外周血作为对照组。

2. 血清miRNAs提取：采集NDMM患者和HD外周血标本，室温下2 000 r/min（离心半径10 cm）离心10 min，分离得到的血清样本按750 µl/份分装，−80 °C保存。取200 µl血清加入1 ml TRIzol涡旋振荡15 s，随后加入200 µl氯仿剧烈振荡，12 000×*g*离心15 min，取水相层，经异丙醇沉淀、75％乙醇洗涤后用15 µl RNAse-free H_2_O重悬。

3. 实时荧光定量PCR（RT-qPCR）检测：方法参考文献[12]，使用1 µg血清RNA进行逆转录（QP016 GeneCopoeia），RT-qPCR检测miR-19a-3p（HmiRQP093 GeneCopoeia）。miR-423-5p（HmiRQP0942 GeneCopoeia）作为RT-qPCR检测内参。RT-qPCR反应条件如下：95 °C 10 min预变性，随后95 °C 10 s，60 °C 20 s，72 °C 10 s进行40个循环扩增。

4. 随访：随访截止时间为2021年2月，中位随访时间45.5（26.8～63.5）个月。总生存（OS）时间定义为自开始治疗至因任何原因死亡或随访终止时间。无进展生存（PFS）时间定义为自开始治疗至疾病进展的时间。

5. 统计学处理：采用SPSS 25.0软件进行统计学分析，约登指数用于确定最佳分界点。计数资料用例数（百分比）表示，计量资料用均数±标准差表示。采用Kaplan-Meier法进行生存分析，组间比较采用Log-rank检验，*P*<0.05为差异有统计学意义。采用Spearman相关性分析。绘制ROC曲线，以灵敏度（真阳性率）为纵坐标，1-特异性（假阳性率）为横坐标，曲线下面积（AUC）决定指标的准确性，左上角最接近顶端的点表示所研究指标的最佳临界值。

## 结果

1. NDMM患者血清中miR-19a-3p的表达水平：RT-qPCR检测201例NDMM患者和60名HD血清标本中的miR-19a-3p。结果显示，与60名HD比较，NDMM患者血清中miR-19a-3p的平均表达水平显著降低［（2.57±2.65）对（4.00±2.53），*P*<0.001］。

2. NDMM患者血清miR-19a-3p表达水平与临床指标的相关性：结果显示，患者血清miR-19a-3p水平与血清白蛋白水平呈负相关（*r*＝−0.205，*P*<0.01），与DS分期、ISS分期、R-ISS分期及患者性别无明显相关性（*P*>0.05）。

3. miR-19a-3p在MM预后评估中的作用：应用ROC曲线分析血清miR-19a-3p水平在MM诊断中的意义，结果显示，AUC为0.662（*P*<0.001），表明miR-19a-3p作为生物标志物可有效区分NDMM患者及HD。应用约登指数计算，血清miR-19a-3p的临界值为2.3505，可根据miR-19a-3p表达水平将NDMM患者分为低表达组（miR-19a-3p≤2.3505，108例）与高表达组（miR-19a-3p>2.3505，93例）。生存曲线结果表明，与高表达组相比，低表达组的PFS时间（22.47个月对38.53个月，*P*＝0.015）和OS时间（77.43个月对未达到，*P*＝0.038）均显著缩短（图1）。

**图1:**
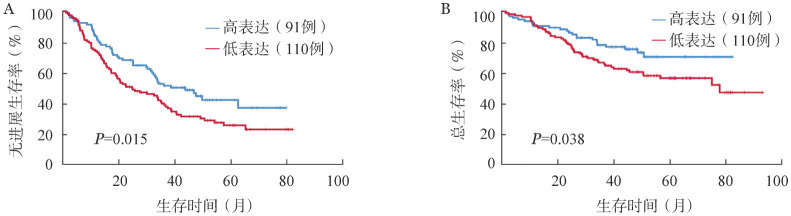
血清miR-19a-3p高表达与低表达组患者的无进展生存（A）及总生存（B）曲线

根据患者的miR-19a-3p水平及治疗方案进行分组，生存分析结果显示，在以硼替佐米为基础的治疗组中，miR-19a-3p低表达组患者的中位PFS时间（19.33个月对33.73个月，*P*＝0.050）及中位OS时间（77.43个月对未达到，*P*＝0.120）较高表达组缩短。在以免疫调节剂为基础的治疗组，miR-19a-3p低表达组患者的中位PFS时间（36.13个月对49.63个月，*P*＝0.176）及中位OS时间（未达到对未达到，*P*＝0.184）较高表达组缩短（图2）。根据患者是否行auto-HSCT及血清中miR-19a-3p的表达水平将患者分为四组。结果显示，血清miR-19a-3p高表达行auto-HSCT组的中位PFS时间（46.7个月对22.47个月，*P*＝0.030）及中位OS时间（未达到对77.43个月，*P*＝0.027）优于miR-19a-3p低表达未行auto-HSCT组（图3）。在miR-19a-3p低表达患者中，行auto-HSCT组患者的OS时间较未行auto-HSCT组延长（未达到对77.43个月，*P*＝0.003）。

**图2:**
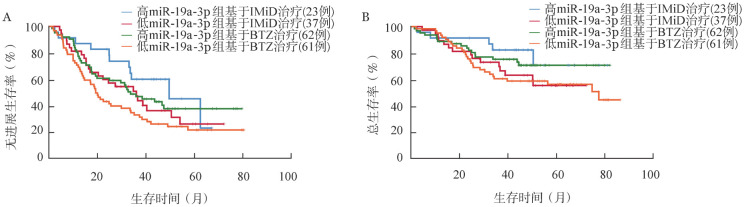
miR-19a-3p高、低表达组患者接受硼替佐米（BTZ）或免疫调节剂（IMiD）治疗的无进展生存（A）及总生存（B）曲线

**图3:**
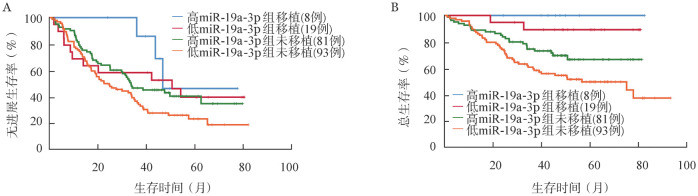
miR-19a-3p高、低表达组患者是否进行自体造血干细胞移植的无进展生存（A）及总生存（B）曲线

## 讨论

虽然MM具有局灶性分布和易伴髓外侵犯等特征，但目前对MM肿瘤负荷的量化评估仍以骨髓为主，因此液体活检在MM分层评估中可能具有特殊的应用价值。液体活检的生物标志物主要包括循环肿瘤细胞（CTC）、循环肿瘤DNA（ctDNA）、细胞外囊泡（EVs，主要是外泌体）、循环无细胞RNA（cfRNA）、miRNA（和cfRNA共同构成了ctRNA）[13]。分析血液中上述癌症组分能够实现肿瘤的早期筛查、分子分型、预后、用药指导及复发监测等。外周血等体液样本较容易获取，大多数情况下，采集过程较组织活检创伤小，更易重复采样。其中miRNAs分子因特异性高、稳定存在于组织液受到众多研究者的关注[14]–[15]。

miRNAs可通过靶向其目的基因促进疾病进展，DIANA-miRPath靶基因通路分析结果显示miR-19a的靶基因是mTOR、p53等信号通路的关键分子，这些信号通路失调与MM细胞增殖活性升高有密切关系[16]。研究发现，骨转移性前列腺癌组织和细胞中miR-19a-3p的表达均明显下调，可通过激活TGF-β信号促进前列腺癌的侵袭、迁移和肿瘤细胞的骨转移[17]。miR-19a也可作为结直肠腺癌、乳腺癌、胃癌等实体肿瘤的生物标志物[18]–[20]。

近十年来，蛋白酶体抑制剂的应用使MM患者的生存期显著延长，平均中位OS时间从2～3年延长至6～7年[21]。因此，在新药时代，miR-19a-3p作为MM诊断及预后标志物是否仍然适用是本研究的目的之一。本研究表明，miR-19a-3p在MM患者中仍然具有辅助诊断价值及评估预后的意义。在201例患者血清标本的研究中，miR-19a-3p能较好地将患者及HD进行区分（AUC＝0.662, *P*<0.001），且NDMM中miR-19a-3p低表达的患者预后较差。基于血清标本易获得的特点，血清循环miRNAs有望应用于肿瘤的早期诊断并在治疗过程中监测肿瘤的动态演变[22]。在miRNAs作为生物标志物应用方面，中山医院研发的“7种微小核糖核酸肝癌检测试剂盒”已得到国家食品药品监管总局的认证并应用于肝癌患者。miR-19a-3p也有望在MM患者早期诊断及预后评估方面应用于临床，本研究组也将致力于MM生物标志物的研究及应用，为患者精准诊断及预后评估提供更切实有效的依据。
